# Temperature-Dependent Activity of Motor Proteins: Energetics and Their Implications for Collective Behavior

**DOI:** 10.3389/fcell.2021.610899

**Published:** 2021-02-26

**Authors:** Saumya Yadav, Ambarish Kunwar

**Affiliations:** Department of Biosciences and Bioengineering, Indian Institute of Technology Bombay, Mumbai, India

**Keywords:** temperature, kinesin, dynein, myosin, arrhenius, molecular motor, intra-cellular transport

## Abstract

Molecular motor proteins are an extremely important component of the cellular transport system that harness chemical energy derived from ATP hydrolysis to carry out directed mechanical motion inside the cells. Transport properties of these motors such as processivity, velocity, and their load dependence have been well established through single-molecule experiments. Temperature dependent biophysical properties of molecular motors are now being probed using single-molecule experiments. Additionally, the temperature dependent biochemical properties of motors (ATPase activity) are probed to understand the underlying mechanisms and their possible implications on the enzymatic activity of motor proteins. These experiments in turn have revealed their activation energies and how they compare with the thermal energy available from the surrounding medium. In this review, we summarize such temperature dependent biophysical and biochemical properties of linear and rotary motor proteins and their implications for collective function during intracellular transport and cellular movement, respectively.

## Introduction

Intracellular transportation system utilizes molecular motor proteins along with their cofactors to haul cargos along cytoskeletal filaments from one location to another. Intra-cellular transport by molecular motor proteins is essential for cell growth, cell division, and cell survival. Most living organisms on earth reside in varying environmental conditions, and some even survive under extreme weather conditions, from boiling water swamps to polar ice desert. Every place on earth is home to different life forms starting from unicellular bacteria and fungi to multicellular vertebrates. Hence, intracellular transport in these organisms is tuned to cater to their survival in adverse and extreme weather conditions. Many biophysical properties of motor proteins are now known to be temperature dependent. Investigations to understand these temperature-dependent properties of motor proteins were initiated from different studies such as understanding of hibernation from the molecular to organism levels under unfavorable environmental conditions (Carey et al., 2003) and temperature-dependent changes in axonal transport (Ochs and Smith, 1971; Grafstein et al., 1972; Heslop and Howes, 1972; Edström and Hanson, 1973; Gross, 1973; Brimijoin, 1974, 1975; Brimijoin and Helland, 1976; Cosens et al., 1976). These studies are the foundational mark to imply that motor properties change due to variation in temperature leading to observed changes in cellular transport, direction, velocity, and other parameters.

The study of thermal properties of a molecular motor initiated from different studies of the cellular phenomenon, for example, a study was conducted on the maintenance of intracellular transport due to unfavorable temperature conditions. They report the attribute of hibernation in mammals follows extended bouts of torpor, during which minimal body temperature (Tb) can fall as low as −2.9°C and metabolism can be reduced to 1% of euthermic rates. It lists down the repetition of survival cycles of torpor and arousal during hibernation period (Truhlar and Kohen, 2001; Carey et al., 2003).

**FIGURE 1:**
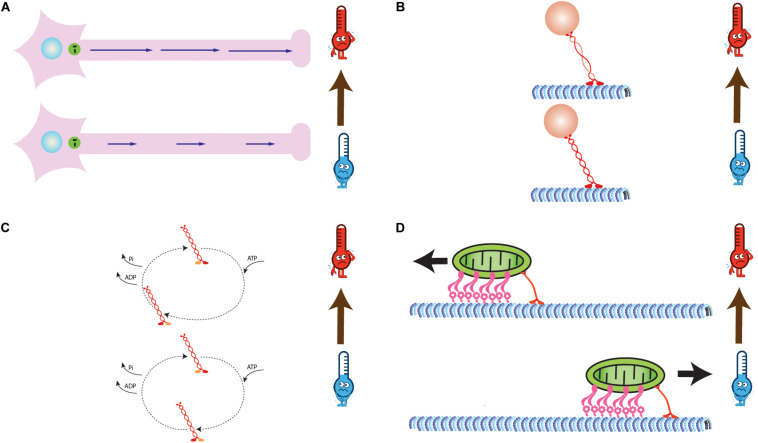
**(A)** Intracellular transport velocity increases with increase in temperature. The length of blue arrows is proportional to magnitude of transport velocities. **(B)** Decrease in helicity of stalk domain of motor proteins with increase in temperature **(C)** Modified biochemical properties of motor proteins (Adenosine triphosphate (ATP) binding, Hydrolysis, and ADP + Pi release rates) due to change in temperature. The size of dashed arrows is proportional to time required for reactions. **(D)** Reversal of direction of bidirectional transport due to change in temperature dependent biophysical properties of a set of antagonistic motors (Kinesin-1 and Dynein).

**FIGURE 2:**
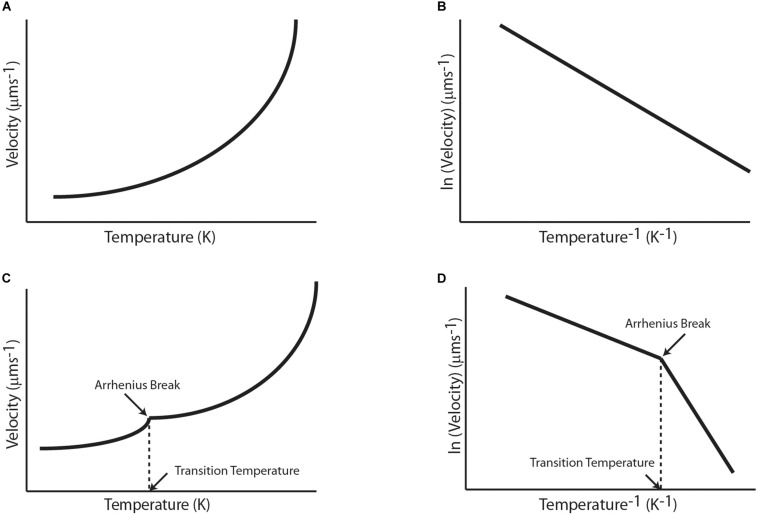
Qualitative graphical representation of reported temperature dependence of biophysical and biochemical properties of molecular motors with temperature for **(A)** A simple Arrhenius enzyme, **(B)** corresponding Arrhenius plot of a simple Arrhenius enzyme, **(C)** A complex Arrhenius enzyme, and **(D)** corresponding Arrhenius plot of a complex Arrhenius enzyme.

Similarly, studies involving axonal transport inside different animal cells have been done to understand their temperature dependent behavior. Such studies involving rabbits and bullfrogs’ sciatic nerves reported an exponential increase in transport velocity of Dopamine-beta-hydroxylase (DBH) with temperature (Figure 1A; Brimijoin, 1974, 1975; Brimijoin and Helland, 1976; Cosens et al., 1976). DBH is a neuronal protein involved in the production of dopamine. Cosens also reported that microtubule density remained mostly stable and decreased only at lower temperatures (∼30%), i.e., below 13°C in rabbit nerves and 10°C in frog nerves (Edström and Hanson, 1973; Cosens et al., 1976). This implies that increase in microtubule density does not significantly affect the transport of DBH along with temperature change. This further implies that exponential increase in velocity is due to changes in inherent properties of molecular motors, which thereafter reflects toward the major conformational change as an underlying mechanism. A separate study involving frog sciatic nerves defines the average transport rate in axonal transport. They report that the transport rate in these cells varies from 32 to 290 mm/day in the temperature range of 5.5–28°C. The temperature coefficient was observed to decrease in this temperature range (3.4–2.3) (Edström and Hanson, 1973). A common finding of these studies reports Arrhenius dependence of transport rates of cellular entities (tagged proteins, macromolecules, etc.) with respect to the temperature inside cells. Arrhenius equation links rates of temperature dependent chemical or physical reactions with respect to Arrhenius constant and Activation energy. Arrhenius constant is the frequency of collisions and depends on the types of molecules involved in the reaction, whereas Activation energy is the energy barrier required by substrates to undergo the reaction. This Arrhenius relation is expressed as-

$$k_{T}=k_{o}*\exp\left(-E_{a}/R\,T\right)$$

where k_*T*_ is kinetic reaction rate, k_*o*_ is Arrhenius constant, E_*a*_ is the activation energy, R is the universal gas constant and T is the absolute temperature (Ashter, 2013). Additionally, some motors exhibit piecewise Arrhenius trend which points toward change in the enzymatic cycle (preferably rate-limiting step) of the motor and are popularly referred to as “Arrhenius Breaks.” Arrhenius Breaks have been observed in various studies on temperature dependent biochemical and biophysical properties of molecular motors. In this review, we first briefly discuss earlier works on temperature dependent transport properties of a tagged protein or cellular entity that were done prior to the classification of motor proteins. These studies used sciatic nerves, olfactory nerve, muscular cells, etc., to study transport rates with different temperature ranges (Yeatman et al., 1969; Gross, 1973; Cosens et al., 1976). The temperature dependence of these rates was evaluated using temperature coefficient (Q_10_) of rates at different temperatures. Q_10_ is a measure of rate of a biological process with temperature difference of 10°. Thereafter, we discuss studies on biochemical properties such as ATPase activity and biophysical properties such as velocity, processivity, and stall force due to variation in temperature for linear motors, respectively. These sections contain studies of Arrhenius dependence and breaks in Arrhenius relationship for Kinesin, Dynein, Myosin, and F1-ATPase motor proteins (Figure 2). Additionally, we discuss studies that hint at the universality of Arrhenius breaks as they have been reported for all kinds of molecular motors (Doval et al., 2020).

## Earlier Studies on Temperature Dependence of Cellular Transport

Cellular transport system of different cell types has roughly the same components, i.e., cargo, motors, and their regulators. Before the discovery of different types of molecular motors, numerous studies on thermal and biochemical properties of cellular environment and transport have been done, which include rates of aggregation, rates of chemical catalysis, average velocity, processivity, etc. These studies date back to the time when cellular motors and their types were unknown; however, they report Arrhenius dependence on temperature indicating toward the role of motors underlying in these observations (Truhlar and Kohen, 2001).

Studies of thermal properties of cellular transport were initially done using sciatic nerves of rabbits, bullfrogs, rats, garfish, etc., and their axonal transport was studied *in vitro* using temperature coefficient Q_10_ (Kawai et al., 2000; Humphries et al., 2003; Grove et al., 2005). Guenter W. Gross in 1973 estimated the temperature dependence of axoplasmic transport rates in the olfactory nerve of garfish through simple distance-time graphs for 10–28°C. His results showed linear dependence of transport rate on temperature with values 53 ± 3.8, 249 ± 5.2, and 410 ± 30 mm/day at 10, 25, and 37°C, respectively (Gross, 1973). In a separate study, rabbit sciatic nerves were observed *in vitro* for axonal transport of dopamine-β-hydroxylase (DBH) activity. DBH was accumulated due to cooling of nerves to 2°C and when this accumulated DBH was subjected to different temperature ranges between 13 and 42°C, the velocity increased exponentially given by relation *V* = 0.546 (1.09)^*T*^**V** = 0.546(1.09)^**T**^, where *V* is velocity of transport in mm/h and *T* is temperature in degree Celsius. The velocity showed Arrhenius dependence on temperature and varies from 0.4 ± 0.1 at 10°C to 12.8 ± 0.6 at 37°C, which again dropped to 1.5 ± 0.9 mm/h at 47°C due to degradation of proteins at such higher temperatures. The temperature coefficient Q_10_ was observed to be 2.33 and activation energy to be 14.8 kcal (Cosens et al., 1976). A study of *in vitro* axonal transport of tagged leucine molecule was observed in frog sciatic nerves from 5.5 to 28°C. The rate of biophysical transport, namely velocity was calculated based on the distance traveled by tagged leucine during the incubation time. They found the rate to increase non-linearly from 32 mm/day at 5.5°C to 290 mm/day at 28°C. Experimentally random values of rates were observed due to instability of protein and its surrounding. However, curve extrapolation yielded a rate to be 400 mm/day approximately. It was suggested that increased electrical activity in the nervous system caused enzymatic changes which alters the metabolic rates in cells (Edström and Hanson, 1973). Mammalian muscle contraction and ATPase cycle rate were studied in rat and mouse muscles’ myofibrils from 8 to 38°C. ATPase rates, rate of increase in tension and maximum velocity had similar Arrhenius temperature dependence with energy of ∼66 kJ/mol. The temperature coefficient (Q_10_) of tension relaxation had a value of 2.5, which means that its rate constant increased 2.5 times with ΔT of 10°C (Stein et al., 1982; Rall and Woledge, 1990). Another study on rat skeletal muscle between 20 and 35°C showed Arrhenius dependence with activation energies to be 40–45 and 60–80 kJ for shortening and relaxation, respectively (Ranatunga, 1982; Roots and Ranatunga, 2008). A study that dates back to 1984 examined force-velocity relationship of fast and slow-twitch muscles in rats in the range of 10–35°C. The curve of force-velocity increased with cooling for both muscles with Q_10_ decreasing by factors of 1.8 from 35 to 25°C and 2.4 below 20°C (Elmubarak and Ranatunga, 1984). Later, the emergence of fluorescence microscopy to study temperature dependent properties of Myosin proteins helped in *in vitro* temperature dependent properties of velocity and processivity of a single motor protein (Anson, 1991; Pierce and Vale, 1998).

Earlier reports on temperature dependent cellular transport were mostly done in terms of transport rate, temperature coefficient Q_10_ and velocity of tagged protein or vesicles transported inside cells. These studies show Arrhenius or exponential dependence of the above listed parameters with temperature. Additionally, temperature coefficient is indicative of underlying transition in their behavior and may be structural or conformational properties too. These properties have been explored in the later studies which we will be discussing in the sections to follow.

## Temperature-Dependent Motor Properties: Arrhenius Relationship and Arrhenius Breaks

### Temperature-Dependent Biochemical Properties of Molecular Motors

ATPase activity is the ability of motor proteins to catalyze the decomposition of ATP. This process is known as ATP hydrolysis and releases the energy which is used for mechanical motion by molecular motors. Temperature dependence of their ATPase activity determines the catalytic efficiency of motor proteins and points towards their confirmational modification as an underlying molecular mechanism as observed in various studies; we will be discussing for both Linear as well as Rotary in subsequent sections.

#### Temperature Dependent ATPase Activity of Linear Motors

Molecular motors derive their energy by catalyzing the decomposition of Adenosine triphosphate (ATP), known as the energy molecule of a living cell. Roughly, a kinesin’s energy efficiency based on the maximum load it can bear (6 pN) is around ∼60%, which is quite higher than any conventional man-made motor. It has been a subject of researchers’ curiosity as to how these motors function in different temperature ranges of cold-blooded or hot-blooded animals and their catalytic efficiency. Temperature dependence of their ATPase activity indicates conformational modification in their head domains or stalk fragment. Hence, these studies focus on either structural modification or rates of catalysis. Temperature dependent *in vitro* studies of different motors reveal Arrhenius-like catalytic behavior of these motors (de Cuevas et al., 1992; Böhm et al., 2000).

Circular Dichroism (CD) based structural analysis of Kinesin from *Drosophila melanogaster* reveals that its stalk fragment is ∼55–60% α-helical at 25°C and 85–90% α-helical at 4°C and 0% β-sheets at either temperature ranges, hence suggesting that stalk fragments of these proteins melt in solution while approaching physiological temperature ranges (de Cuevas et al., 1992). Further, they report that coil 1 of Kinesin stalk is thermally more stable as compared to coil 2 (Figure 1B). This might regulate the binding properties of kinesin. However, based on their observation, they conclude that stalk may regulate force production *in vivo*, but it does not affect the *in vitro* motility of Kinesin and Myosin motor (Toyoshima et al., 1987; Lovell et al., 1988; de Cuevas et al., 1992). Additionally, they report that Kinesin motor protein undergoes conformational modification which breaks its Arrhenius dependence into two major temperature ranges, i.e., 25–30 and 45–50°C. Hence, it indirectly reflects that conformational change might be the underlying molecular mechanism for piecewise Arrhenius trend for molecular motors. It can be inferred from these observations that conformational changes lead to a change in enzymatic reaction times (rate-limiting step) and rates (Figure 1C), hence the trend (de Cuevas et al., 1992). However, this study did not evaluate temperature dependent transport properties of Kinesin motor protein (no subtypes of Kinesin were discovered back them). A study involving gliding motility assay of kinesin for temperatures between 5 and 37°C explored the effect of conformational changes on kinesin gliding velocity and ATPase activity. Both, the gliding velocity and ATPase activity showed Arrhenius dependence on temperature with a break at 27°C recorded for both and activation energies to be 79 and 5 kJ/mol for temperature range above and below 27°C. Gliding velocity and ATPase activity have a similar profile and are linearly correlated and increased up to 37°C. However, beyond this range, MTs detach from the surface rigorously, which leads to decrease in gliding velocity and ATPase activity. A linear correlation between gliding velocity and ATPase activity in this study suggests an efficient motor system utilizing chemical energy for mechanical force generation. Through this study, the authors studied correlation between ATPase activity and motor processivity to know if a step in ATPase cycle is rate-limiting and responsible for similar thermal dependence of velocity. The correlation plot revealed linear curve between velocity and ATPase activity. This curve can be used to qualitatively estimate the efficiency of conversion of chemical energy to mechanical energy by Kinesin (Böhm et al., 2000).

Another study on kinesin-3 motor protein derived from *T. lanuginosis* estimates its ATP hydrolysis rate with respect to temperature and ATP concentration and was reported to follow Michaelis-Menten kinetics with change in ATP concentration, while it was found to be constant, i.e., ∼1 s^–1^ (non-coupling between ATPase rate and gliding velocity) with temperature. Authors claim that this is due to turnover rate of the reaction to be below signal-to-noise threshold of the assay since gliding motility assay show Arrhenius behavior in this temperature range. Hydrolysis rate displayed a sharp increase from 45 to 65°C with maximum values in the range of 60–65°C (∼6 s^–1^) (Rivera et al., 2007).

#### Temperature-Dependent ATPase Activity of Rotary Motors

F_1_-ATPase is a rotary motor protein which couples ATP hydrolysis to mechanical rotations. They are membrane bound motors which either use NADH coupled energy to make ATP energy molecules (mitochondria) or use ATP hydrolysis for rotational motion and act as channels of uptake or release (cell membrane). They make rotations in steps of 120°. Temperature dependence of their ATPase activity and torque generation has been speculated upon with different approaches. An experimental study of mitochondrial membrane composition on ATPase activity was done in *Saccharomyces cerevisiae* containing different concentrations of ergosterol (7.3 mg/g protein) showed not only Arrhenius dependence on temperature but breaks in this behavior too with transition temperature decreasing from 34 to 18°C as the sterol content increased from 7.3 to 105 mg of ergosterol/g protein. This early study reflects that it can be used to tune the ATPase activity of mitochondrial F_1_-ATPase motor protein (Cobon and Haslam, 1973). Another study involving F_1_ rotary motor shows Arrhenius Break at 17°C with the increase in Activation Energy and points toward its conformational change with temperature (Baracca et al., 1986). Analysis of Bovine-heart F1-ATPase using circular dichroism showed conformational changes in the protein which resulted in a break in the Arrhenius plot with 2.7-fold increase in ATP hydrolysis activation energy. Earlier studies along with the present study show temperature dependence of ATP hydrolysis activity and the same also being the rate determining step for ATPase activity of F_1_ rotary motor. Although, transition temperature may vary for motors derived from different sources, the conformational change was observed in all of them nevertheless (Baracca et al., 1989). However, these studies do not report reaction intermediate and rate-limiting step in the ATPase activity of F_1_-ATPase. In the subsequent work, the analyses of F1-ATPase rotation at different temperature values (2, 4, 9, and 23°C) showed ADP release to be an intermediate step and also temperature dependent. Hence, it would competitively suppress the rate of ADP release through negative feedback by regulating the concentration of free ADP in the solution. Additionally, they reported an unusually high Q_10_ factor of 19. This could be due to high conformational change in the protein with an increase in temperature (Watanabe et al., 2008). Another study on beef mitochondrial F1 (thermophilic F1) shows that it remains active in the range of 0–90°C with ADP release as the intermediate temperature dependent step.

F1-ATPase is a mixture of an active and an inhibited domain. After the addition of nucleotide-free F_1_, most of the F1 molecules are active. However, at higher temperatures, comparatively fewer F1 molecules are active with 35°C becoming an equilibrium mixture of active and inhibited populations. This is self-explanatory as its physiological role is ATP synthesis and not ATP hydrolysis or its consumption and thermophilic F1 is stable with its ATPase activity peak at 70°C. The rates of F1 rotation increase with temperature which is expected for any protein until it denatures (Furuike et al., 2008).

All the linear or rotary motors follow Arrhenius dependence on temperature of their catalytic behavior and have been speculatively suggested to undergo structural modifications or conformational changes. This modification is more evident around the transition temperature which results in the change in activation energy causing Arrhenius Breaks. Some *in vitro* studies focus on attaining thermal stability of motors and study different domains to understand which of them plays a crucial role in their function and processivity. Studies done so far reveal that the stalk fragments or the coils of motors are temperature sensitive and undergo major modifications. It might be due to their maximum exposure to the surface in 3D conformation of the motor.

### Temperature-Dependent Biophysical Properties of Motors

Molecular motors harness chemical energy from ATP energy packets to perform mechanical motion such as translation, rotation, or sliding. Motors entail certain characteristic properties that govern the mechanical behavior of motors. These are categorized as biophysical properties of motors. Processive or linear motors carry cellular bodies from one place to another in a directed manner by trailing on cytoskeletal filaments (actin and microtubules). Their biophysical properties include rates of attachment, stepping, and detachment on and from their tracks, processivity, and velocity. Processivity is the total distance traveled by a motor before detaching from its track. On the other hand, rotary motors’ rates of rotation are defined in terms of the rotation steps of 120°. Rotary motors are embedded in the bi-layer membranes of cells and membranous organelles of the cells. F1-ATPase is a rotary motor that drives the electron pump to generate ATP energy molecules.

#### Temperature-Dependent Biophysical Properties of Linear Motors

Motor proteins are involved in specific transport mechanism inside cells, for example, cytoplasmic Dynein and Kinesin motors working in groups inside cells, Myosin II involved in muscle contraction and relaxation (Howard, 1996; Hirokawa, 1998; Kato et al., 1999). Hence, all the motors have unique biophysical properties according to their function. Additionally, Kinesin and Dynein being antagonistic pair of motors constantly undergo tug-of-war to haul cargo (bodies that are transported by motors inside cells such as mitochondria) to their desired locations. Some studies have also reported sliding mechanism as a means of cargo transport. This majorly happens for non-processive motors which are tethered and the cargo body slides over them (Stepp et al., 2017).

Force-velocity relationship defines the load-bearing capacity of a motor (Svoboda and Block, 1994). Kinesin motors have been most extensively studied motors due to their stability in different *in vitro* experimental conditions. Kawaguchi in 2000 used gliding assay to estimate velocity and run-length, and bead assay to estimate the velocity of a single kinesin (Sakowicz et al., 1999). Gliding assay and bead assay are two *in vitro* assays to study different properties of a single motor under a microscope (Howard et al., 1989; Block et al., 1990). In bead assay, stabilized microtubules are tethered to glass surfaces and motors are in the solution, where as in gliding assay, motors are tethered to the glass while microtubules slide over them (Yamashita et al., 1994). Kinesin motor’s velocity was reported to increase with Arrhenius activation energy of 50 kJ/mol and average run-length of the motor increased qualitatively from ∼0.7 to ∼1.5 μm between 15 and 35°C. Increased processivity implies decreased possibility of detachment of kinesin with increasing temperature. However, to author’s surprise, generated force remained unchanged thermally with an average value of ∼7 pN. Based on the findings, force generation was coupled with nucleotide-binding state or conformational change in kinesin, whereas velocity was coupled with temperature-sensitive ATPase activity. Hence, both the properties are coupled with different steps of mechanochemical cycle of kinesin motor. They also studied the force-velocity relation at different temperatures and the curve was linear for all temperature ranges. Thermal tuning of velocity without change in its force generation capacity determines the efficiency of motors to utilize the chemical energy derived from ATP molecules in their mechanical motion (Svoboda and Block, 1994; Kawaguchi and Ishiwata, 2000). However, Shin’ichi Ishiwata in his repeated studies for different temperature ranges did not report any breaks in the temperature dependent Arrhenius relation of Kinesin motor protein. They estimated temperature dependent biophysical properties between 15 and 35°C for kinesin in 2000. Later in 2001, they used temperature pulse microscopy (TPM) which helps to elevate temperatures till the boiling temperatures to analyze the same properties at higher temperatures, as the proteins used to degrade at higher temperatures earlier. It uses illuminating thin metal layer, evaporating it on the glass surface with infrared laser beam. The illumination is shut off and on periodically and heat dissipates into the surrounding medium in 10 ms. It allows to record motor activity at elevated temperature by activating it thermally. TPM was used by Kawaguchi to estimate the gliding velocities of MTs at temperatures higher than 35°C up to 53°C. The average velocities were obtained from 3.65 μm/s at 50°C to 0.48 μm/s at 20°C. However, repeated thermal activation damaged kinesin molecules and acted as an internal load for their movement. They obtained the velocity values within the temperature range of 15 and 35°C and the curve fitting was done with no Arrhenius breaks, activation energy being 50 kJ/mol (Kawaguchi and Ishiwata, 2001). They did not record any Arrhenius breaks in this study too (Kawaguchi and Ishiwata, 2000, 2001). However, in 2006, a quantitative estimation of run-length, duration, and velocity was done using single kinesin isolated from bovine brain between 20 and 40°C. This group recorded deviation of linear Arrhenius velocity curve above 30°C with activation energy to be 48 kJ/mol, which is quite close to the earlier recorded values, i.e., transition temperature to be 27°C and activation energy 50°C. However, they base these findings on the deterioration of proteins at elevated temperatures, and hence, the less steep slopes with slower rates of ATP hydrolysis and decreased velocity. Additionally, detachment probability was calculated indirectly from one cycle of kinesin stepping (8 nm). Average run-length was reported to increase from 0.6 to 1.3 μm and average duration decreased from 1.5 to 0.7 s at 20 and 40°C, respectively. On the other hand, velocity followed a Gaussian distribution at each temperature, increasing exponentially following Arrhenius relation with temperature. The activation energy was found to be 48 kJ/mol (in accordance with the previous value, i.e., 50 kJ/mol). Detachment probability decreased with temperature from 0.0143 at 20°C to 0.0062 at 40°C. Here, temperature dependence of run-length is weaker than velocity as duration is inversely proportional to temperature and run-length is the dependent parameter among all three of them (Nara and Ishiwata, 2006). However, all of these studies have been done for dimeric kinesin. Thermal biochemical and biophysical properties of monomeric protein, i.e., Kinesin-3 were studied by the research group of George D. Bachand. They studied temperature dependent gliding motility rates and ATP hydrolysis rate for kinesin-3 (TKIN) isolated from *Thermomyces lanuginosus* (fungus). Apart from temperature dependence, gliding velocity was observed with ATP concentration and followed Michaelis-Menten kinetics with the change in ATP concentration. The gliding velocity displayed Arrhenius dependence on temperature with maximum velocity of 5.5 μm/s at 45°C. MTs depolymerized beyond this temperature range and failed to bind to tethered kinesin molecules. The Arrhenius energy (∼103 kJ mol^–1^) is quite high and almost twice than those reported for Eg5 or ncd-like kinesin (Crevel et al., 1997). Hence, authors conclusively state that TKIN belongs to thermostable enzymes as they display increased Arrhenius energies. Additionally, they reported the break both for ATP hydrolysis as well as gliding velocity for Kinesin-3 *in vitro* at 27°C. Their recorded range is as high as 65°C for ATP hydrolysis, whereas 45°C for velocity (Rivera et al., 2007). Beyond this range, microtubules become highly unstable and do not bind to kinesin-coated surfaces which results in a sharp decrease in velocity. Interestingly, in this study both the activities follow similar Arrhenius behavior above 20°C (Aquilanti et al., 2010). Below this range, ATP hydrolysis curve goes flat. This might be due to turnover rate being in the range of noise as stated earlier. Surprisingly, the Kinesin-3 seems to be the fastest motor at physiological temperature range (Rivera et al., 2007; Hong et al., 2016) with the velocity of 5.5 μm/s. Despite this, its turnover rate is low. This discrepancy may be due to motors being less processive and non-cooperative to haul cargo (Rivera et al., 2007).

Apart from the Kinesin superfamily, cytoplasmic Dynein is also a processive motor that transports cellular entities from cell periphery to the nucleus. Additionally, these motors are also a part of the flagellum in cells and enable their beating to help the cells move in a defined gradient of a micronutrient. An *in vitro* single molecule-based study was conducted on the velocity of mammalian Kinesin-1, mammalian cytoplasmic Dynein and yeast cytoplasmic Dynein. Mammalian cytoplasmic Dynein showed the break at 15°C, yeast Dynein showed the break at ∼8°C, whereas Kinesin-1 showed no breaks. This implies that Dynein motors are thermally sensitive motors, hence more tunable. This study emphasized on the tuning of the net direction of cargo transport by varying number of Dynein and Kinesin motors, respectively. Since transport inside cells is carried out by a team of different kinds of similar as well as antagonistic motors, hence this sheds light at more realistic picture of intracellular transport. The net transport direction of a cargo is determined by the biophysical properties of each type of engaged motors in the team. Additionally, tuning of these rates can be done by varying the number or physiological conditions of the surrounding environment. This can help change the net direction of transport, run-length as well as velocity of both unidirectional and bidirectional transport. In the temperature-dependent study of Kinesin-1 and Dynein, the velocity and detachment rate of Kinesin-1 and mammalian cytoplasmic Dynein were found to follow Arrhenius relation with temperature. The velocity of Dynein exhibited a break from simple Arrhenius relation at 15°C, whereas Kinesin-1 showed no such behavior. On the other hand, yeast cytoplasmic Dynein showed this break at ∼8°C. Additionally, they put forth temperature dependent mathematical expression of detachment rate, velocity, and run length of single Kinesin-1 and mammalian Dynein motors. Extremely high activation energy below 15°C for Dynein means that Dynein is more sensitive to temperature, shuts down at low temperatures, and hence it is thermally more tunable, whereas Kinesin-1 is a relatively thermostable motor. Hence, in bidirectional transport governed by Dynein and Kinesin-1 motors in the ratio of 4:1, cargo tends to move in the negative direction of MTs at higher temperatures (>15°C), whereas at lower temperature range, it moves in the positive direction (Figure 1D; Hong et al., 2016). Hence, Kinesin-1 dominates in lower temperature ranges. Study of temperature dependent biophysical properties of Kinesin-1 motor protein *in vitro* has always been limited below 30°C due to its degradation. However, such is not the case inside a cell as it functions well at temperatures close to 40°C.

Next, there is a class of axonemal Dynein motors present in beating flagellum which are responsible for their periodic beating with microtubules sliding over the tethered motors. This environment is similar to *in vitro* gliding assay, i.e., Dynein motors inside flagellum function in a similar set-up. This explains the Arrhenius dependence of flagellum’s oscillation frequency in the observed temperature range of 5–35°C (Machemer, 1972; Coakley and Holwill, 1974; Mitchell, 2007; Warren et al., 2010). Flagellum oscillation frequencies showed breaks at 17°C. These locomotive organelles contain tethered Dynein motors which slide microtubules in a periodic manner and facilitate the beating of the flagellum. Hence, Dynein motors are responsible for spontaneous oscillations (SO) and the underlying drivers for piecewise Arrhenius dependence. Corresponding activation energies are 30 and 43 kJ mol^–1^ above and below 17°C (Warren et al., 2010). The SO frequency (*w(T)*) was expressed as a function of temperature as ln(*w(T*)) = $\frac{-E}{R\,T}+\text{ln}\,\left(K\right)$, where *T* is the temperature, *E* is activation energy, *K* is pre-exponential factor, and *R* is the gas constant.

On the other hand, the study of F-actin sliding on skeletal myosin *in vitro* between 3 and 42°C shows change in its velocity from 11 nms^–1^ at 3°C to 12 μms^–1^ at 42°C. Additionally, their experiments showed the same curve for both cooling and heating indicating reversibility of the temperature dependent rates. However, Arrhenius break at 15.4°C and activation energies are reported to be 50 and 289 kJ/mol for temperatures >15and <15°C, respectively. The energy for lower temperature range is abnormally higher. This indicates that Myosin might have a different rate-limiting step unlike other molecular motors due to drastic difference in activation energies of both the ranges. Additionally, the temperature dependence of ATP hydrolysis and velocity of Myosin motors above 20°C are similar but different at lower temperatures (Anson, 1991; Higuchi and Goldman, 1991). Detection of transport velocity of rhodamine phalloidin-labeled F-actin moving *in vitro* on rabbit skeletal myosin revealed a non-linear Arrhenius plot by M. Anson in 1992 (Anson, 1991). The curve was fitted to follow a cubic relation with temperature. Additionally, the author report same profile for both cooling and heating which indicates reversibility in the rates of enzymatic behavior of motors. However, the curve was divided in two linear ranges with activation energies to be 50 kJ/mol above 15°C and 289 kJ/mol below it with corresponding temperature coefficients (Q_10_) to be 1.9 and 76.5. This study reports three orders of magnitude increase in actin velocity. However, results differ at lower temperatures, i.e., the curve follows a linear relation roughly (Anson, 1991). Hence, this study provides evidence that temperature dependence of velocity and ATP hydrolysis might be uncoupled.

Briefly, so far temperature-based studies of motors recorded breaks from Arrhenius activity for Dynein and Myosin motors from different sources, namely, Drosophila, mammals, rat brain sciatic nerves, etc. (Edström and Hanson, 1973; Gross, 1973; Cosens et al., 1976; Highsmith, 1977; Stein et al., 1982; Rall and Woledge, 1990). However, for Kinesin motor proteins, studies contradicted each other in reporting deviations or breaks. It has been reported that Kinesin motors obtained from different sources show deviations at different temperatures. *Drosophila* Kinesin-1 shows transition at 17°C, whereas bovine brain Kinesin-1 reported this transition at 27°C (Crevel et al., 1997; Böhm et al., 2000). Research group of M. Vershinin introduced TMAO as the heat-protecting agent to prevent degradation of Kinesin-1 *in vitro.* 200 mM TMAO successfully stabilized the kinesin till 50°C (Chase et al., 2017). Although, it has been long known that velocity follows a Gaussian distribution with peak at the mean velocity at a fixed temperature, the same was demonstrated for Kinesin-1 and Kinesin-3 (KIF1A) (Chase et al., 2017; Doval et al., 2020). Furthermore, single-molecule *in vitro* experiments on Kinesin-1 and Kinesin-3 motility reveal Arrhenius dependence of velocity on temperature both in the presence of ATP as well as GTP. However, average velocity decreased with the use of GTP as an energy molecule and the Activation energy for GTP was higher (Doval et al., 2020). The main focus of this work was to study Arrhenius breaks for Kinesin motor proteins. They report break for Kinesin-1 at 4.7°C and for Kinesin-3 at 10.5°C. These transition temperature values clarify the disputed breaks for Kinesin motor proteins, i.e., they fall outside the physiological range and hence were uncovered in the temperature regimes taken before. Additionally, they observed that these transition temperatures can be varied from 10.5 to 16°C for Kinesin-3 by using 200 mM TMAO *in vitro* (Ma et al., 2014; Doval et al., 2020). A review done in 2003 focused on the maintenance of intracellular transport due to unfavorable temperature conditions. They report the attribute of hibernation in mammals follows extended bouts of torpor, during which minimal body temperature (Tb) can fall as low as −2.9°C and metabolism can be reduced to 1% of euthermic rates (Carey et al., 2003).

Transport and motor properties discussed above often showed Arrhenius dependence on temperature and sometimes a piecewise Arrhenius-trend, i.e., the linearity of Arrhenius plot breaks at a transition temperature which divides the curve in two range of temperatures with distinct activation energies.

#### Temperature-Dependent Biophysical Properties of Rotary Motors

As discussed earlier, rotary motors are known to undergo drastic conformational modification which alters their ATPase activity. Additionally, these studies explore their rotational motion as a function of ATP concentration as well as temperature. The research group of Hiroyuki Noji in 2008 reported that rotational rate followed Michaelis–Menten relation with ATP concentration at 23 and 4°C. Similarly, the rates increased slightly with temperature from 4 to 23°C (Watanabe et al., 2008). In a different study, rotations of Beef heart F1-ATPase were recorded in the temperature range of 4–40°C. The rates were observed in distinct steps of 120° at saturating ATP concentrations (2 mM) and were observed to increase with temperature with Arrhenius dependence. Apparently, they did not observe any breaks in this behavior and ADP release was observed to be rate-limiting step (Furuike et al., 2008). Another study by Hiroyuki Noji focused exclusively on biophysical properties of F1 rotary motion and used magnetic tweezers to understand temperature dependence of rotational torque by analyzing temperature sensitive (TS) reaction (intermediate reaction of ADP release) and hydrolysis dwell times. They studied both k^*on*^ and k^*off*^ rates of TS reaction signifying forward and reverse reaction. K^*on*^ increased exponentially by approximately 6.2-fold per 20°C, whereas k^*off*^ remained constant at 0.3 s^–1^. This implies strong temperature sensitivity of rotation at all temperature ranges (Watanabe and Noji, 2014). Quite recently, the effects of temperature on the angular velocity profile of the 120°F_1_-ATPase power stroke were resolved at a time resolution of 10 μs (Martin et al., 2018). The angular velocity of F_1_-ATPase changed inversely with temperature during phase 1 (0°–60°) with a parabolic dependence resulting in negative activation energy values. This directly indicates that it was powered by elastic energy of a torsional spring consistent with unwinding the γ-subunit coiled-coil. In contrast, Phase 2 of the power stroke had an enthalpic component suggesting that additional energy input was required to enable the γ-subunit to overcome energy stored by the spring after rotating beyond its equilibrium position. The correlation between the probability distribution of ATP binding to the empty catalytic site and the negative activation values of the power stroke during phase 1 indicates that this additional energy is derived from the binding of ATP to the empty catalytic site. Based on these observations, an elastic coupling mechanism was proposed that uses the coiled-coil domain of the γ-subunit rotor as a torsion spring during phase 1, and as a crankshaft which powered by ATP-binding–dependent conformational changes during phase 2 to drive the power stroke (Martin et al., 2018).

The indirect implication of studies discussed above on linear and rotary motors is that not only Arrhenius relationship but also break in this profile is a characteristic behavior of molecular motors as they undergo conformational change. However, the scale of this modification can vary over different motor families or source organisms determining the transition temperature, thermal stability, or sensitivity of these biomolecules.

## Discussion

In this review, we briefly list and discuss the major findings of temperature dependent kinetics of molecular motors and the energies involved, the abruptness or the abnormal behavior observed and the underlying molecular mechanisms. We started with preliminary studies of pre-motor era when different types of molecular motor proteins and their possible roles were not known. These research publications focused straightaway on successfully varying surrounding temperature of the sample and studying the transport rates of tagged proteins (DBH, leucine, etc.) inside the cells (Gross, 1973; Cosens et al., 1976). Others focused on understanding the rates of tension (extension and contraction) in muscle cells *in vitro* (Stein et al., 1982; Rall and Woledge, 1990). Additionally, the temperature dependence of rates was evaluated in terms of temperature coefficient Q_10_ which revealed the ratio of rates at the difference of 10°C.

Temperature change tends to modify conformation of both linear as well as rotary motor proteins. These modifications have been presumed to be responsible for change in enzymatic activity of motors, hence changing their rates of ATPase activity in ATP hydrolysis. On the other hand, it is perceived that modified rates of ATP hydrolysis reflect in the duration of each step in the motor stepping cycle. Hence, influencing the properties of velocity, runlength, force production, etc. Therefore, both mechanochemical and biophysical properties of motors have been studied with variation in temperature. With the study of structure of kinesin motor protein, it was found that one of the two domains of its stalk fragment are less stable with temperature which leads to the change in binding properties of kinesin with temperature. In the next series of studies, attempts were being made to unwind any hidden correlation between Arrhenius relation of ATPase activity and velocity of kinesin. However, different temperature regimes were followed in these studies. One of the studies reported linear curve of correlation between the two phenomena (27–35°C), whereas the other study reported deviation of ATP-hydrolysis curve from linear Arrhenius behavior below 20°C. This was, however, attributed to lower turnover rate of the studied kinesin-3 motor protein and different kinesin proteins used in these studies (Crevel et al., 1997). Rotary motors on the other hand show temperature dependence due to ADP release step which is said to be its temperature-sensitive (TS) reaction. Similar to linear motors, they also follow Arrhenius relationship with temperature but their Q_10_ factor is unusually high (17) due to high conformational change. However, this is only speculative theory and has not been subjected to experimental validation or *in silico* simulation studies on 3-D protein conformation.

The advancement of single-molecule experiments, optical trap, temperature pulse microscopy (TPM) and Fluorescence Total internal reflection microscopy (FTIR) facilitated *in vitro* studies of biophysical properties of molecular motors and their subtypes. Bead assay and gliding assay are two kinds of *in vitro* methods to study transport properties of motors under different physiological conditions. Hence, temperature dependent biophysical parameters, i.e., Force generation, run-length, velocity, and rate of detachment are studied using these assays (Mao et al., 2005). A series of *in vitro* studies focused on the behavior of motor velocity, its run-length and force production capacity with different ranges of temperatures. Interestingly, velocity and run-length increased exponentially following Arrhenius relation with temperature, but force generation capacity of motors remained unchanged (average force ∼7 pN for Kinesin). This discrepancy is attributed to force generation being dependent on temperature-independent nucleotide-binding step whereas velocity being dependent on temperature-dependent ATP hydrolysis cycle. Force-velocity curve followed linear relation at different temperatures (Kawaguchi and Ishiwata, 2000). The ability of motor to translate the generated force into its processivity shows its efficiency. Hence, the slope of force-velocity curve at different temperatures qualitatively reflects the efficiency of that motor. Studies of kinesin motors revealed maximum efficiency of 60% for these nanomachines in the physiological temperature range. Molecular motors are also present in beating organelle-flagellum of a cell. Spontaneous oscillation (SO) frequencies of flagellum were reported to show Arrhenius dependence on temperature. This is reflective of temperature sensitivity of Dynein motors present in these organelles (Warren et al., 2010). Furthermore, this study also explicitly recorded the break in temperature dependent curve at 17°C. However, it cannot be conclusively stated that this is due to property of engaged Dynein motors in flagellum. But later, in a follow up, *in vitro* single-molecule experiment of mammalian and yeast Dynein revealed break at ∼15 and 8°C (Hong et al., 2016). Kinesin-1 on the other hand did not show any breaks in the observed temperature range. However, they not only studied temperature dependence of biophysical properties of single motor molecules *in vitro*. They also put forth theoretical expressions for temperature dependence of detachment rate, stepping rate, velocity, and run-length for Dynein and Kinesin-1 motor protein. Furthermore, they also explained physical effects of these properties on bidirectional transport by the group of these antagonistic motors. Dynein is thermally more sensitive whereas Kinesin-1 is the thermally stable motor. Thus, Dynein motors in the ratio of 4:1 with Kinesin-1 leads the cargo in negative direction with increase in temperature, whereas Kinesin-1 motors dominate the transport at lower temperature ranges and haul the cargo in positive direction of microtubules (Figure 1D). These observations are reflective of the concentration, roles, and positioning of these motors in cellular transport of organisms with different body temperatures and requirements (warm-blooded mammals, cold-blooded reptiles, ectotherms, etc.). In another study, gliding of F-actins over Myosin proteins had shown the break at 5°C. Hence until recently, it was assumed that the property of Arrhenius breaks was limited to Dynein and Myosin motors, but Arrhenius breaks observed for Kinesin-1 and Kinesin-3 at ∼4 and 10°C show that motors of Kinesin superfamily follow piecewise Arrhenius trend too. Thus, it can be stated that Arrhenius breaks are universal to the behavior of molecular motors and can be tuned by varying the chemical concentration of motor proteins and energy molecules (Chase et al., 2017; Doval et al., 2020).

## Author Contributions

SY and AK wrote the manuscript together. Both authors contributed to the article and approved the submitted version.

## Conflict of Interest

The authors declare that the research was conducted in the absence of any commercial or financial relationships that could be construed as a potential conflict of interest.

## References

[B1] Anson M. (1991). Temperature dependence of the velocity of fluorescently labelled F-actin sliding on rabbit skeletal myosin in-vitro. *J. Physiol*. 224, 1029–1038. 10.1016/0022-2836(92)90467-x 1533250

[B2] Aquilanti V. Mundim K. C. Elango M. Kleijn S. Kasai T. (2010). Temperature dependence of chemical and biophysical rate processes: phenomenological approach to deviations from Arrhenius law. *Chem. Phy. Lett*. 498 209–213. 10.1016/j.cplett.2010.08.035

[B3] Ashter S. A. (2013). “Mechanics of materials,” in *Thermoforming of Single and Multilayer Laminates (1st ed.)*, ed. Elsevier D. S. E. (Elsevier: William Andrew Publications), 123–145. 10.1016/B978-1-4557-3172-5.00006-2

[B4] Baracca A. Amler E. Solaini G. Parenti Castelli G. Lenaz G. Houstek J. (1989). Temperature-induced states of isolated F1-ATPase affect catalysis, enzyme conformation and high-affinity nucleotide binding sites. *Biochim. Biophys. Acta Bioenerg*. 976 77–84. 10.1016/s0005-2728(89)80191-4 2527562

[B5] Baracca A. Curatola G. Castelli G. P. Solaini G. (1986). The kinetic and structural changes of the mitochondrial F1-ATPase with temperature. *Biochem. Biophy. Res. Commun*. 136 891–898. 10.1016/0006-291X(86)90416-X 2872889

[B6] Block S. M. Goldstein L. S. Schnapp B. J. (1990). Bead movement by single kinesin molecules studied with optical tweezers. *Nature* 348 348–352. 10.1038/348348a0 2174512

[B7] Böhm K. J. Stracke R. Baum M. Zieren M. Unger E. (2000). Effect of temperature on kinesin-driven microtubule gliding and kinesin ATPase activity. *FEBS Lett*. 466 59–62. 10.1016/s0014-5793(99)01757-3 10648812

[B8] Brimijoin S. (1974). Local changes in subcellular distribution of dopamine-beta-hydroxylase (EC 1.14.2.1) after blockade of axonal transport. *J. Neurochem*. 22 347–353. 10.1111/j.1471-4159.1974.tb07599.x 4829961

[B9] Brimijoin S. (1975). Stop-flow: a new technique for measuring axonal transport, and its application to the transport of dopamine-beta-hydroxylase. *J. Neurobiol*. 6 379–394. 10.1002/neu.480060404 52690

[B10] Brimijoin S. Helland L. (1976). Rapid retrograde transport of dopamine-beta-hydroxylase as examined by the stop-flow technique. *Brain Res*. 102 217–228. 10.1016/0006-8993(76)90878-7 55294

[B11] Carey H. V. Andrews M. T. Martin S. L. (2003). Mammalian hibernation: cellular and molecular responses to depressed metabolism and low temperature. *Physiol. Rev*. 83 1153–1181. 10.1152/physrev.00008.2003 14506303

[B12] Chase K. Doval F. Vershinin M. (2017). Enhanced stability of kinesin-1 as a function of temperature. *Biochem. Biophys. Res. Commun*. 493 1318–1321. 10.1016/j.bbrc.2017.09.172 28986254

[B13] Coakley C. J. Holwill M. E. (1974). Effects of pressure and temperature changes on the flagellar movement of *Crithidia oncopelti*. *J. Exp. Biol*. 60 605–629. 4847274 10.1242/jeb.60.3.605

[B14] Cobon G. S. Haslam J. M. (1973). The effect of altered membrane sterol composition on the temperature dependence of yeast mitochondrial ATPase. *Biochem. Biophys. Res. Commun*. 52 320–326. 10.1016/0006-291x(73)90990-x 4268187

[B15] Cosens B. Thacker D. Brimijoin S. (1976). Temperature-dependence of rapid axonal transport in sympathetic nerves of the rabbit. *J. Neurobiol*. 7 339–354. 10.1002/neu.480070406 60464

[B16] Crevel I. M. Lockhart A. Cross R. A. (1997). Kinetic evidence for low chemical processivity in ncd and Eg5. *J. Mol. Biol*. 273 160–170. 10.1006/jmbi.1997.1319 9367754

[B17] de Cuevas M. Tao T. Goldstein L. S. (1992). Evidence that the stalk of *Drosophila kinesin* heavy chain is an alpha-helical coiled coil. *J. Cell Biol*. 116 957–965. 10.1083/jcb.116.4.957 1734025 PMC2289341

[B18] Doval F. Chiba K. McKenney R. J. Ori-McKenney K. M. Vershinin M. D. (2020). Temperature-dependent activity of kinesins is regulable. *Biochem. Biophys. Res. Commun*. 528 528–530. 10.1016/j.bbrc.2020.05.157 32507595 PMC7366363

[B19] Edström A. Hanson M. (1973). Temperature effects on fast axonal transport of proteinsin vitro in frog sciatic nerves. *Brain Res*. 58 345–354. 10.1016/0006-8993(73)90006-1 4127873

[B20] Elmubarak M. H. Ranatunga K. W. (1984). Temperature sensitivity of tension development in a fast-twitch muscle of the rat. *Muscle Nerve* 7 298–303. 10.1002/mus.880070408 6727914

[B21] Furuike S. Adachi K. Sakaki N. Shimo-Kon R. Itoh H. Muneyuki E. (2008). Temperature dependence of the rotation and hydrolysis activities of F1-ATPase. *Biophy. J*. 95 761–770. 10.1529/biophysj.107.123307 18375515 PMC2440441

[B22] Grafstein B. Forman D. S. McEwen B. S. (1972). Effects of temperature on axonal transport and turnover of protein in goldfish optic system. *Exp. Neurol*. 34 158–170. 10.1016/0014-4886(72)90196-3 4109685

[B23] Gross G. W. (1973). The effect of temperature on the rapid axoplasmic transport in C-fibers. *Brain Res*. 56 359–363. 10.1016/0006-8993(73)90353-3 4123713

[B24] Grove T. J. McFadden L. A. Chase P. B. Moerland T. S. (2005). Effects of temperature, ionic strength and pH on the function of skeletal muscle myosin from a eurythermal fish, *Fundulus heteroclitus*. *J. Muscle Res. Cell Motil*. 26 191–197. 10.1007/s10974-005-9010-0 16179972

[B25] Heslop J. P. Howes E. A. (1972). Temperature and inhibitor effects on fast axonal transport in a molluscan nerve. *J. Neurochem*. 19 1709–1716. 10.1111/j.1471-4159.1972.tb06215.x 4114446

[B26] Highsmith S. (1977). The effects of temperature and salts on myosin subfragment-1 and F-actin association. *Arch. Biochem. Biophys*. 180 404–408. 10.1016/0003-9861(77)90054-6 879794

[B27] Higuchi H. Goldman Y. E. (1991). Sliding distance between actin and myosin filaments per ATP molecule hydrolysed in skinned muscle fibres. *Nature* 352 352–354. 10.1038/352352a0 1852212

[B28] Hirokawa N. (1998). Kinesin and dynein superfamily proteins and the mechanism of organelle transport. *Science* 279 519–526. 10.1126/science.279.5350.519 9438838

[B29] Hong W. Takshak A. Osunbayo O. Kunwar A. Vershinin M. (2016). The effect of temperature on microtubule-based transport by cytoplasmic Dynein and Kinesin-1 motors. *Biophy. J*. 111 1287–1294. 10.1016/j.bpj.2016.08.006 27653487 PMC5034348

[B30] Howard J. (1996). The movement of kinesin along microtubules. *Annu. Rev. Physiol*. 58 703–729. 10.1146/annurev.ph.58.030196.003415 8815816

[B31] Howard J. Hudspeth A. J. Vale R. D. (1989). Movement of microtubules by single kinesin molecules. *Nature* 342 154–158. 10.1038/342154a0 2530455

[B32] Humphries M. M. Kramer D. L. Thomas D. W. (2003). The role of energy availability in mammalian hibernation: an experimental test in free-ranging eastern chipmunks. *Physiol. Biochem. Zool*. 76 180–186. 10.1086/367949 12794671

[B33] Kato H. Nishizaka T. Iga T. Kinosita K. Ishiwata S. (1999). Imaging of thermal activation of actomyosin motors. *Proc. Natl. Acad. Sci. U.S.A*. 96 9602–9606. 10.1073/pnas.96.17.9602 10449739 PMC22255

[B34] Kawaguchi K. Ishiwata S. (2000). Temperature dependence of force, velocity, and processivity of single kinesin molecules. *Biochem. Biophy. Res. Commun*. 272 895–899. 10.1006/bbrc.2000.2856 10860848

[B35] Kawaguchi K. Ishiwata S. (2001). Thermal activation of single kinesin molecules with temperature pulse microscopy. *Cell Motil. Cytoskeleton* 49 41–47. 10.1002/cm.1019 11309839

[B36] Kawai M. Kawaguchi K. Saito M. Ishiwata S. (2000). Temperature change does not affect force between single actin filaments and HMM from rabbit muscles. *Biophys. J*. 78 3112–3119. 10.1016/S0006-3495(00)76848-2 10827988 PMC1300893

[B37] Lovell S. Karr T. Harrington W. F. (1988). Suppression of contractile force in muscle fibers by antibody to myosin subfragment 2. *Proc. Natl. Acad. Sci. U.S.A*. 85 1849–1853. 10.1073/pnas.85.6.1849 2964637 PMC279878

[B38] Ma J. Pazos I. M. Gai F. (2014). Microscopic insights into the protein-stabilizing effect of trimethylamine N-oxide (TMAO). *Proc. Natl. Acad. Sci. U.S.A*. 111 8476–8481. 10.1073/pnas.1403224111 24912147 PMC4060645

[B39] Machemer H. (1972). Temperature influences on ciliary beat and metachronal coordination in *Paramecium*. *J. Mechanochem. Cell Motil*. 1 57–66.

[B40] Mao H. Arias-Gonzalez J. R. Smith S. B. Tinoco I. Bustamante C. (2005). Temperature control methods in a laser tweezers system. *Biophys*. *J*. 89 1308–1316. 10.1529/biophysj.104.054536 15923237 PMC1366615

[B41] Martin J. L. Ishmukhametov R. Spetzler D. Hornung T. Frasch W. D. (2018). Elastic coupling power stroke mechanism of the F 1 -ATPase molecular motor. *Proc. Natl. Acad. Sci. U.S.A.* 115 5750–5755. 10.1073/pnas.1803147115 29760063 PMC5984535

[B42] Mitchell D. R. (2007). The evolution of eukaryotic cilia and flagella as motile and sensory organelles. *Adv. Exp. Med. Biol*. 607 130–140. 10.1007/978-0-387-74021-8-11 17977465 PMC3322410

[B43] Nara I. Ishiwata S. (2006). Processivity of kinesin motility is enhanced on increasing temperature. *Biophysics* 2 13–21. 10.2142/biophysics.2.13 27857556 PMC5036643

[B44] Ochs S. Smith C. (1971). “Effect of temperature and rate of stimulation on fast axoplasmic transport in mammalian nerve fibers,” in *Federation Proceedings* (Bethesda MD), 30, A665.

[B45] Pierce D. W. Vale R. D. (1998). Assaying processive movement of kinesin by fluorescence microscopy. *Methods Enzymol*. 298 154–171. 10.1016/s0076-6879(98)98016-8 9751879

[B46] Rall J. A. Woledge R. C. (1990). Influence of temperature on mechanics and energetics of muscle contraction. *Am. J. Physiol*. 259(2 Pt 2) R197–R203. 10.1152/ajpregu.1990.259.2.R197 2201213

[B47] Ranatunga K. W. (1982). Temperature-dependence of shortening velocity and rate of isometric tension development in rat skeletal muscle. *J. Physiol*. 329 465–483. 10.1113/jphysiol.1982.sp014314 7143257 PMC1224791

[B48] Ranatunga K. W. (1984). The force-velocity relation of rat fast- and slow-twitch muscles examined at different temperatures. *J. Physiol*. 351 517–529. 10.1113/jphysiol.1984.sp015260 6747875 PMC1193132

[B49] Rivera S. B. Koch S. J. Bauer J. M. Edwards J. M. Bachand G. D. (2007). Temperature dependent properties of a kinesin-3 motor protein from *Thermomyces lanuginosus*. *Fungal. Genet. Biol*. 44 1170–1179. 10.1016/j.fgb.2007.02.004 17398126

[B50] Roots H. Ranatunga K. W. (2008). An analysis of the temperature dependence of force, during steady shortening at different velocities, in (mammalian) fast muscle fibres. *J. Muscle Res. Cell Motil*. 29 9–24. 10.1007/s10974-008-9138-9 18523851 PMC2493522

[B51] Sakowicz R. Farlow S. Goldstein L. S. (1999). Cloning and expression of kinesins from the thermophilic fungus *Thermomyces lanuginosus*. *Protein Sci*. 8 2705–2710. 10.1110/ps.8.12.2705 10631986 PMC2144245

[B52] Stein R. B. Gordon T. Shriver J. (1982). Temperature dependence of mammalian muscle contractions and ATPase activities. *Biophys. J*. 40 97–107. 10.1016/S0006-3495(82)84464-0 6216923 PMC1328982

[B53] Stepp W. L. Merck G. Mueller-Planitz F. Ökten Z. (2017). Kinesin-2 motors adapt their stepping behavior for processive transport on axonemes and microtubules. *EMBO Rep*. 18 1947–1956. 10.15252/embr.201744097 28887322 PMC5666610

[B54] Svoboda K. Block S. M. (1994). Force and velocity measured for single kinesin molecules. *Cell* 77 773–784. 10.1016/0092-8674(94)90060-4 8205624

[B55] Toyoshima Y. Y. Kron S. J. McNally E. M. Niebling K. R. Toyoshima C. Spudich J. A. (1987). Myosin subfragment-1 is sufficient to move actin filaments in vitro. *Nature* 328 536–539. 10.1038/328536a0 2956522

[B56] Truhlar D. Kohen A. (2001). Convex Arrhenius plots and their interpretation. *Proc. Natl. Acad. Sci. U.S.A*. 98 848–851. 10.1073/pnas.98.3.848 11158559 PMC14672

[B57] Warren B. Lukashkin A. N. Russell I. J. (2010). The dynein-tubulin motor powers active oscillations and amplification in the hearing organ of the mosquito. *Proc. Biol. Sci*. 277 1761–1769. 10.1098/rspb.2009.2355 20129974 PMC2871864

[B58] Watanabe R. Iino R. Shimabukuro K. Yoshida M. Noji H. (2008). Temperature-sensitive reaction intermediate of F1-ATPase. *EMBO Rep*. 9 84–90. 10.1038/sj.embor.7401135 18064048 PMC2246616

[B59] Watanabe R. Noji H. (2014). Characterization of the temperature-sensitive reaction of F1-ATPase by using single-molecule manipulation. *Sci. Rep*. 4:4962. 10.1038/srep04962 24825532 PMC4019956

[B60] Yamashita H. Sata M. Sugiura S. Momomura S. Serizawa T. Iizuka M. (1994). ADP inhibits the sliding velocity of fluorescent actin filaments on cardiac and skeletal myosins. *Circ. Res*. 74 1027–1033. 10.1161/01.res.74.6.1027 8187272

[B61] Yeatman L. A. Parmley W. W. Sonnenblick E. H. (1969). Effects of temperature on series elasticity and contractile element motion in heart muscle. *Am. J. Physiol*. 217 1030–1034. 10.1152/ajplegacy.1969.217.4.1030 5824301

